# Clinical Epigenomics in Rare Diseases: Interpreting DNA Methylation Episignatures

**DOI:** 10.1002/mgg3.70319

**Published:** 2026-10-02

**Authors:** Himanshu Goel, Shuxiang Goh, Luke Stuart

**Affiliations:** ^1^ Hunter Genetics Waratah New South Wales Australia; ^2^ University of Newcastle Callaghan New South Wales Australia; ^3^ School of Clinical Medicine University of New South Wales Sydney New South Wales Australia; ^4^ Manchester University NHS Foundation Trust Manchester UK

**Keywords:** chromatinopathy, clinical epigenomics, DNA methylation, episignature, long‐read sequencing, m6A, multi‐omics, neurodevelopmental disorder, rare disease, variant of uncertain significance

## Abstract

**Background:**

Epigenomic testing complements sequence‐based analysis by detecting downstream changes in epigenomic state associated with genetic variation. Genome‐wide DNA methylation episignatures are reproducible molecular phenotypes that can serve as biomarkers of specific Mendelian disorders, particularly those involving chromatin regulators, DNA methylation machinery, and transcriptional regulatory pathways.

**Methods:**

We reviewed the biological basis, laboratory methodology, analytical approaches and clinical applications of DNA methylation episignature testing, with emphasis on neurodevelopmental disorders and rare diseases. We also considered current computational tools, limitations of clinical interpretation and emerging epigenomic and epitranscriptomic approaches.

**Results:**

DNA methylation episignature testing is now used clinically to support molecular diagnosis, assist interpretation of variants of uncertain significance and distinguish overlapping neurodevelopmental and chromatin‐related disorders. Interpretation integrates methylation‐array data, statistical and machine‐learning classification, phenotype, genotype and assay‐specific validation. Important limitations include tissue specificity, mosaicism, developmental effects, incomplete disorder coverage and dependence on reference datasets. Emerging approaches include tissue‐agnostic classifiers, long‐read methylation profiling, additional epigenomic signatures and multi‐omic integration.

**Conclusion:**

DNA methylation episignatures provide a clinically useful functional layer of evidence by detecting downstream epigenomic consequences of genomic variation. They should be interpreted as an adjunct to sequence‐based diagnosis and clinical assessment rather than as a replacement for either. Continued expansion of reference datasets and integration with other functional genomic approaches should broaden their diagnostic utility.

## Graphical abstract

Clinical epigenomics extends rare‐disease diagnostics beyond DNA sequence by revealing the downstream regulatory consequences of genetic variation. DNA methylation episignatures provide functional evidence that can support diagnosis, refine variant interpretation, distinguish overlapping disorders, and complement genomic, and multi‐omic testing.

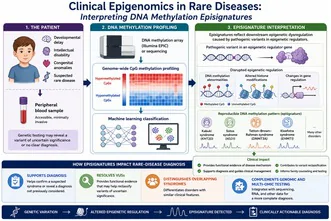

## 1 Introduction

The diagnostic evaluation of developmental delay, intellectual disability, autism spectrum disorder, epilepsy, congenital anomalies and rare diseases has changed profoundly with chromosomal microarray, exome sequencing and genome sequencing (van der Sanden et al. 2023). These technologies have improved the detection of copy‐number variants, sequence variants and structural alterations. They have shortened the diagnostic odyssey for many families (AlAbdi et al. 2023). Nevertheless, a substantial proportion of patients remain without a definitive molecular diagnosis, and many reported variants remain clinically uncertain even after genome‐scale testing.

The residual diagnostic gap reflects more than missed coding variants. Some pathogenic mechanisms occur in non‐coding regulatory elements, repetitive regions, complex structural rearrangements, imprinted loci or regions that are difficult to align with short reads (AlAbdi et al. 2023). Others alter splicing, allele‐specific expression (Dingemans et al. 2022), chromatin state, DNA methylation (LaFlamme et al. 2024), RNA stability or translation in a tissue‐specific or developmental‐stage‐specific manner. Mosaic variation may be below the detection threshold of standard assays or absent from the tissue tested (Francis et al. 2023). These mechanisms create situations in which the sequence result alone is insufficient to establish causality.

Clinical genomic practice is therefore moving from sequence‐only interpretation toward mechanism‐aware functional diagnostics (Lawrence et al. 2026). DNA methylation profiling, transcriptome sequencing, long‐read sequencing and multi‐omic integration are increasingly used to interrogate molecular consequences that are not captured by variant detection alone (Audet et al. 2023; Kernohan and Boycott 2024; Krgovic et al. 2026). DNA methylation episignature testing is one of the most clinically mature epigenomic approaches (Kerkhof et al. 2024). It is particularly relevant to disorders of chromatin regulation, where a pathogenic variant can alter the genome‐wide regulatory state and generate a reproducible methylation pattern detectable in peripheral blood (Haghshenas et al. 2021). The term *episignature* is used here generically to describe a reproducible disease‐associated epigenomic pattern and should not be conflated with EpiSign, the trademarked clinical DNA methylation assay developed by the Western University/London Health Sciences Centre group (Aref‐Eshghi et al. 2019).

An episignature is best understood as a molecular phenotype rather than as a primary genetic lesion. It does not usually identify the causal variant directly; instead, it reflects the downstream epigenomic consequences of disrupted gene regulation. In this sense, sequencing identifies candidate DNA variants, whereas episignature testing evaluates whether those variants, or an unresolved disease mechanism, have produced a methylation profile consistent with a recognised disorder. This distinction is essential for clinical interpretation because a methylation episignature provides functional evidence of disease mechanism, not direct variant detection (Kerkhof et al. 2024; Sadikovic et al. 2021).

Epigenetic disorders are not simply disorders of methylation quantity; they are disorders of the systems that write, erase, read, and propagate regulatory marks on DNA, histones, and RNA. This review places DNA methylation episignatures within the broader framework of clinical epigenomic diagnostics, with emphasis on neurodevelopmental and rare diseases.

## 2 Epigenetic Regulation as Writer, Eraser and Reader Biology

The writer–eraser–reader framework provides a useful structure for understanding epigenetic regulation as shown in Figure 1. Writers install regulatory marks, erasers remove them, and readers interpret them by recruiting downstream molecular machinery. Pathogenic variants can affect any component of this system. A disorder may result from failure to deposit an activating mark, inability to remove a repressive mark, inappropriate recognition of methylated DNA, disrupted chromatin remodelling, or altered interpretation of modified RNA. Among epigenetic modifications, DNA methylation is the most mature and widely implemented biomarker in clinical diagnostics (Frauer et al. 2011). Table 1 compares the clinical relevance of DNA methylation writers, erasers, and readers.

**FIGURE 1:**
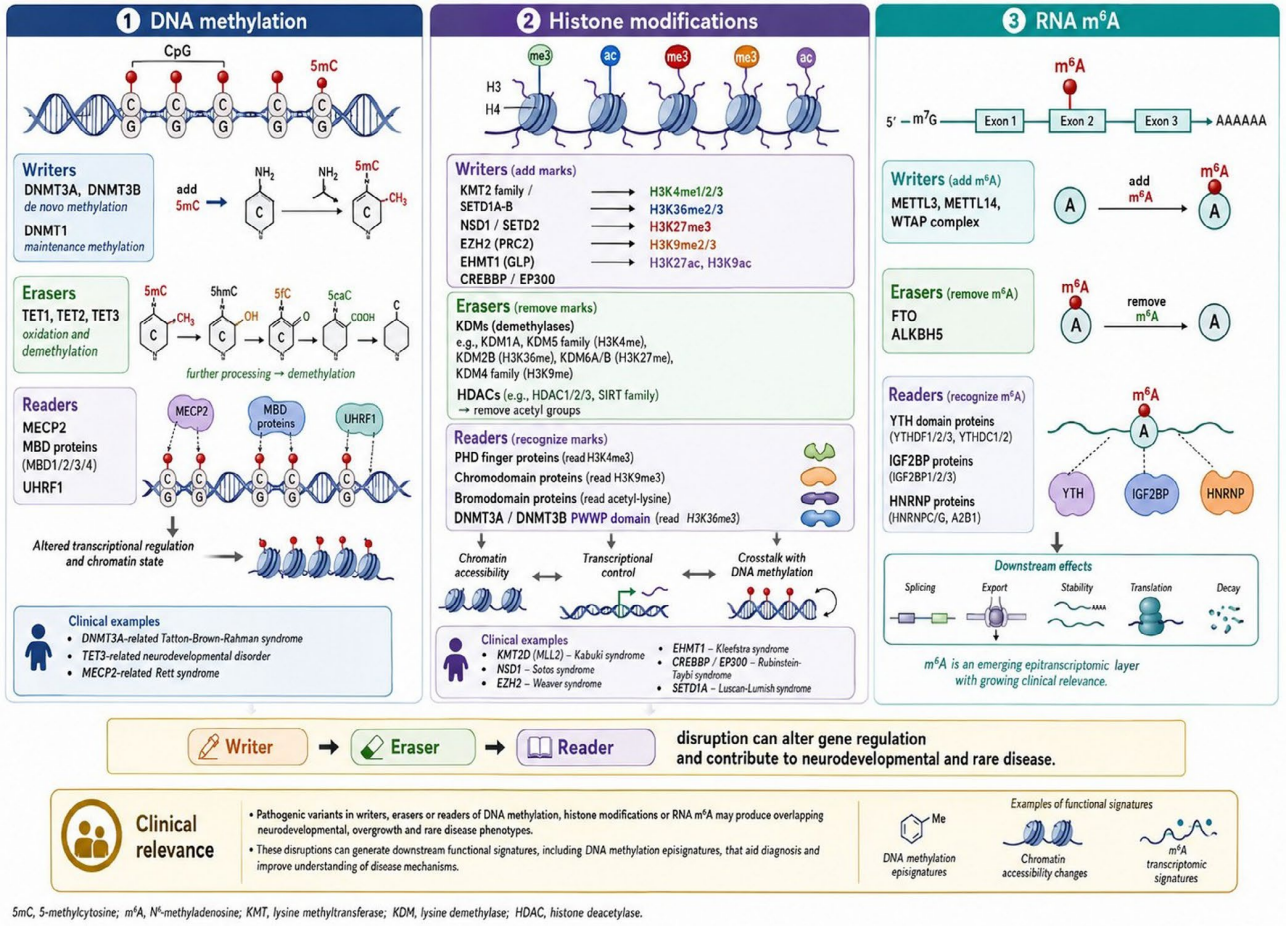
Writer‐eraser‐reader framework in clinical epigenomics. (1) DNA methylation marks are written by DNMT enzymes, modified through TET‐mediated demethylation pathways and interpreted by methyl‐CpG binding proteins and related readers. (2) Histone marks are deposited by methyltransferases and acetyltransferases, removed by demethylases and deacetylases, and recognised by reader domains such as PHD, chromodomain, bromodomain and PWWP modules. (3) RNA m6A is deposited by the METTL3‐METTL14‐WTAP writer complex, removed by FTO and ALKBH5, and interpreted by YTH, IGF2BP and HNRNP reader proteins. Disruption of any layer can alter gene regulation and contribute to neurodevelopmental or rare disease phenotypes.

**TABLE 1:** Writer‐eraser‐reader systems relevant to clinical epigenomics.

Regulatory layer	Writer examples	Eraser/modifier examples	Reader examples	Clinical examples/relevance
DNA methylation (5mC)	DNMT1, DNMT3A, DNMT3B	TET1, TET2, TET3 through 5mC oxidation and repair/replication‐linked replacement	MECP2, MBD and UHRF1 proteins	DNMT3A‐related Tatton‐Brown‐Rahman syndrome (MIM #615879); TET3‐related NDD (MIM #312750); MECP2‐related Rett syndrome (MIM #312750); imprinting disorders
H3K4 methylation	KMT2 family, SETD1A/B	KDM1A/KDM5 families	PHD‐domain proteins, CHD proteins	Kabuki syndrome 1 (MIM #147920); KDM5C‐related X‐linked intellectual disability (MIM #300534); promoter/enhancer regulation
H3K9 methylation	EHMT1, EHMT2, SUV39H1, SUV39H2	KDM3/KDM4 families	HP1 proteins	Kleefstra syndrome (MIM #610253); heterochromatin maintenance and transcriptional repression
H3K27 methylation	EZH2, PRC2	KDM6A, KDM6B	CBX/PRC1 proteins	Weaver syndrome; Kabuki type 2 (MIM #300867); Polycomb dysregulation
H3K36 methylation	NSD1, NSD2, NSD3, SETD2	KDM2/KDM4 families	DNMT3A and DNMT3B PWWP domains, LEDGF	Sotos syndrome (MIM #117550); SETD2‐related Luscan‐Lumish syndrome (MIM #616831); gene‐body DNA methylation
Histone acetylation	EP300, CREBBP, KAT6A, KAT6B	HDACs, sirtuins	Bromodomain proteins	Rubinstein‐Taybi syndrome (MIM #180849) and (MIM #613684); KAT6A/KAT6B‐related disorders
RNA m6A	METTL3‐METTL14‐WTAP complex; VIRMA, RBM15, RBM15B, ZC3H13 as regulatory cofactors	FTO, ALKBH5	YTHDF/YTHDC proteins, IGF2BP proteins, selected HNRNP proteins	Emerging epitranscriptomic mechanism; not yet routine for most rare‐disease diagnostics

Pathogenic variants in *DNMT3A* cause Tatton‐Brown‐Rahman syndrome, a neurodevelopmental overgrowth disorder with a recognisable methylation profile (Kumps et al. 2023). TET3‐related neurodevelopmental disorder illustrates a disease caused by the disruption of cytosine oxidation and demethylation pathways (Levy et al. 2021). MECP2‐related Rett syndrome demonstrates how altered reading and interpretation of methylated DNA can cause severe neurodevelopmental disease (Watson et al. 2026).

Histone modifications add a second regulatory layer. H3K4 methylation is generally associated with active promoters and enhancers and is deposited by KMT2‐family and SETD1‐family methyltransferases (Yu and Lesch 2024). It is removed by demethylases such as KDM1A and the KDM5 family, while plant homeodomain fingers, chromodomains and other reader modules recognise these states. Pathogenic variants in *KMT2D* and *KDM6A* cause Kabuki syndrome 1 and Kabuki syndrome 2, respectively, while *KDM5C* variants cause X‐linked intellectual disability with a characteristic methylation profile in affected males and, in some studies, female carriers (Khodaeian et al. 2021).

Repressive chromatin states are also clinically relevant. H3K9 methylation is associated with heterochromatin and transcriptional repression (Yabe et al. 2024). EHMT1 and EHMT2 deposit H3K9 methylation in euchromatic regions, while SUV39H1 and SUV39H2 contribute to constitutive heterochromatin; HP1 proteins act as key readers of H3K9 methylation. EHMT1‐related Kleefstra syndrome illustrates the clinical consequence of disturbed heterochromatin regulation (Kleefstra et al. 2012). H3K27 methylation, deposited by PRC2 components including EZH2 and removed by KDM6A and KDM6B, regulates Polycomb‐mediated repression and developmental gene control, with variants contributing to overgrowth, undergrowth, and malformation syndromes (Cyrus et al. 2019).

H3K36 methylation is particularly important for linking histone marks to DNA methylation. NSD1 deposits H3K36me2, while SETD2 deposits H3K36me3 (Shipman et al. 2024). These marks are associated with transcribed gene bodies and are recognised by PWWP domains in DNMT3A and DNMT3B (Qiu et al. 2002). In broad terms, H3K36me2 helps target DNMT3A to chromatin domains, whereas H3K36me3 is strongly associated with DNMT3B recruitment to active gene bodies (Qiu et al. 2002; Taglini et al. 2024). Pathogenic *NSD1* variants in Sotos syndrome and *SETD2* variants in Luscan‐Lumish syndrome demonstrate how disruption of H3K36 methylation can produce broad methylation changes and overlapping overgrowth‐neurodevelopmental phenotypes (Douglas et al. 2003; Luscan et al. 2014).

Histone acetylation differs from methylation in that it directly modifies the electrostatic interaction between histones and DNA. Acetylation of lysine residues neutralises positive charge, reducing histone–DNA affinity and generally promoting chromatin accessibility and transcriptional activation. The enzymes responsible for depositing, removing and recognising acetylation marks follow the writer–eraser–reader paradigm. Key acetyltransferase writers include EP300, CREBBP, KAT6A and KAT6B; erasers include histone deacetylases and sirtuins; and readers include bromodomain‐containing proteins. The clinical importance of this regulatory system is illustrated by disorders such as Rubinstein–Taybi syndrome and KAT6A‐ and KAT6B‐related syndromes. Notably, KAT6B‐associated disorders demonstrate distinct DNA methylation episignatures that correlate with underlying molecular mechanisms (Aref‐Eshghi et al. 2020). Variants causing Genitopatellar syndrome (GPS) (MIM #606170), typically truncating changes in the 3′ region of exon 18 that are predicted to escape nonsense‐mediated decay, are associated with a distinct episignature consistent with a probable gain‐of‐function mechanism. In contrast, variants causing Say‐Barber‐Biesecker‐Young‐Simpson syndrome (SBBYSS) (MIM #603736), usually truncating variants located more proximally within exon 18 or earlier in the gene and predicted to result in loss of function, exhibit a separate episignature, highlighting the ability of methylation profiling to distinguish mechanistically distinct disorders arising from the same gene. Genes encoding epigenetic writers, erasers and readers function upstream of chromatin organisation and transcriptional regulation. Disruption of these regulators can produce coordinated methylation changes across thousands of CpG sites, generating the characteristic episignatures observed in many chromatin disorders.

## 3 DNA Methylation Episignatures: Definition and Biological Interpretation

Early studies demonstrated that pathogenic variants in certain chromatin‐regulating genes produce distinct and highly reproducible DNA methylation patterns in peripheral blood, providing the first evidence that disruption of epigenetic regulatory pathways could generate measurable downstream molecular phenotypes (Figure 2). Subsequent investigations expanded the catalogue of recognised episignatures, improved their sensitivity and specificity, and enabled clinical implementation through large reference cohorts, curated episignature databases, and machine‐learning–based classification frameworks.

**FIGURE 2:**
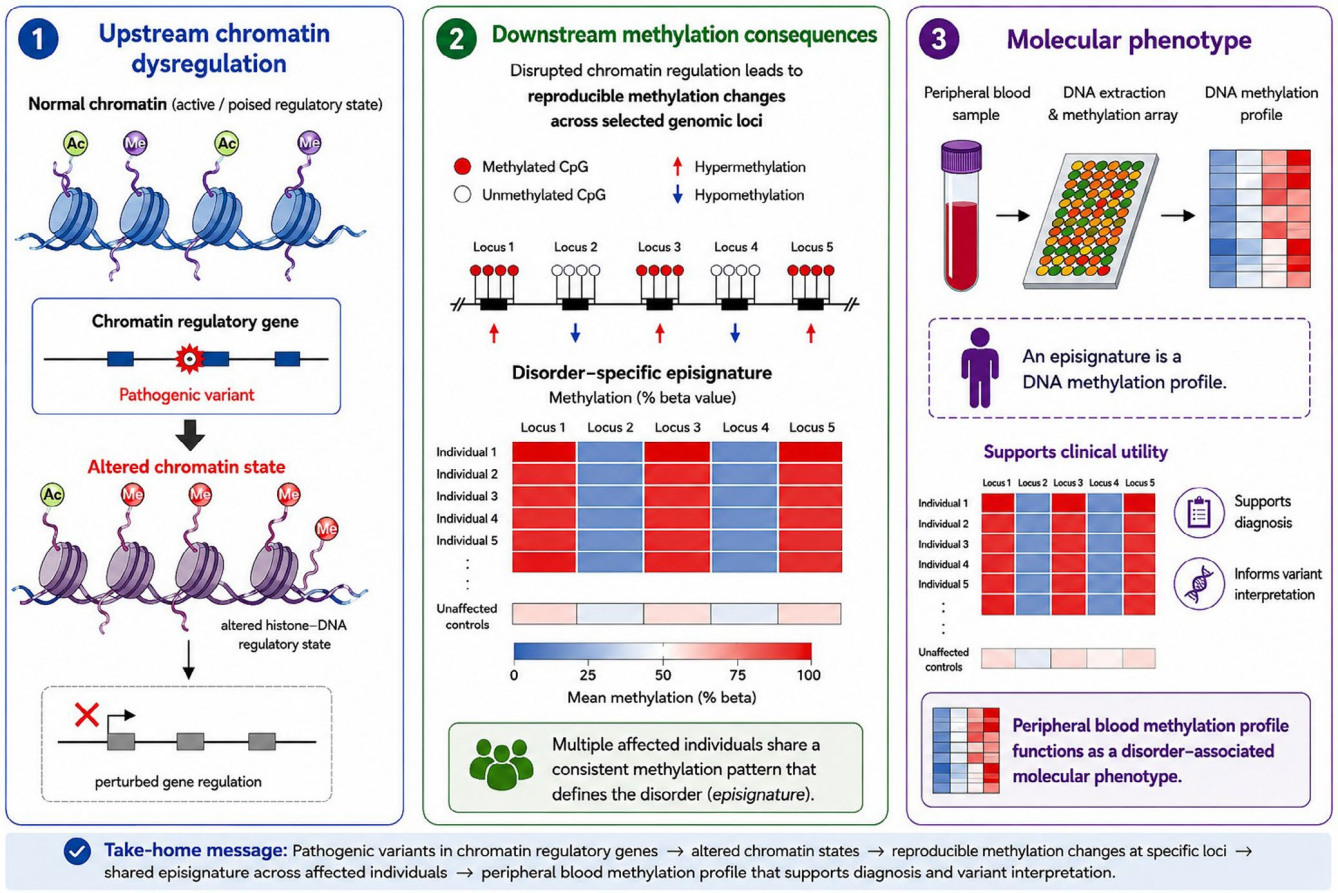
DNA methylation episignatures as downstream molecular phenotypes. (1) Pathogenic variants in chromatin regulatory genes alter histone–DNA regulatory states and perturb gene regulation. (2) Disrupted chromatin regulation can produce reproducible hypermethylation and hypomethylation changes across selected genomic loci. When shared across affected individuals, these changes define a disorder‐associated DNA methylation episignature. (3) Peripheral‐blood methylation profiling can therefore function as a molecular phenotype in selected conditions, supporting diagnosis, variant interpretation and differentiation of clinically overlapping syndromes.

The development of DNA methylation episignatures has involved independent groups and analytical frameworks. The Western University/London Health Sciences Centre group developed EpiSign, a clinically implemented genome‐wide DNA methylation assay and associated classification framework (Aref‐Eshghi, Bend, et al. 2019). Independently, the Weksberg group at The Hospital for Sick Children and the University of Toronto developed EpigenCentral, a platform for analysing genome‐wide DNA methylation data and classifying rare neurodevelopmental disorders (Awamleh et al. 2022; Turinsky et al. 2020). Although both approaches use disorder‐associated methylation patterns for sample classification, they differ in their reference datasets, feature‐selection strategies, classifier implementation, and reporting frameworks. Consequently, classifier outputs should be interpreted according to the validation and performance characteristics of the specific platform rather than assumed to be directly interchangeable.

The biological specificity of an episignature reflects the genomic targets, developmental timing, tissue distribution and molecular function of the affected regulator. Disruption of *NSD1* alters H3K36‐associated methylation patterns and produces a hypomethylation signature that supports Sotos syndrome diagnosis (Choufani et al. 2015). Disruption of *KDM5C*, a histone demethylase acting on H3K4 methylation, produces a distinct peripheral‐blood methylation pattern (Grafodatskaya et al. 2013). Variants in DNA methylation machinery, such as *DNMT3A*, generate different profiles (Kumps et al. 2023). Thus, episignatures are not generic markers of neurodevelopmental disability; they are disorder‐associated patterns arising from specific perturbations of regulatory networks.

The scope of episignature analysis is also expanding beyond classical neurodevelopmental chromatinopathies. A recent study identified a distinct DNA methylation episignature in Diamond–Blackfan anemia syndrome (DBAS; OMIM phenotypic series PS105650), a genetically heterogeneous ribosomopathy and inherited bone‐marrow‐failure disorder, demonstrating that reproducible methylation phenotypes can arise from disease mechanisms outside canonical epigenetic regulatory pathways (Quarello et al. 2026).

Although most clinically characterised episignatures have been developed for Mendelian disorders, reproducible methylation patterns can also reflect environmental exposures. Prenatal alcohol exposure provides an important example. Early epigenome‐wide studies identified differential DNA methylation associated with fetal alcohol spectrum disorder, and more recent work has defined a peripheral‐blood episignature for clinically confirmed fetal alcohol syndrome with potential utility as a supportive molecular biomarker. This broadens the concept of an episignature beyond monogenic disease while also emphasising that such profiles should complement, rather than replace, established clinical diagnostic criteria (Portales‐Casamar et al. 2016; van der Laan et al. 2025).

Clinical utility is strongest in three settings. Firstly, episignature testing can support the reclassification of a variant of uncertain significance in a gene with a validated episignature. Secondly, it can help resolve phenotypically suggestive but sequence‐unsolved cases when the relevant disorder has a validated episignature. Thirdly, it can discriminate between clinically overlapping syndromes that sit in the same regulatory pathway but have different episignatures. Recent clinical series and reporting recommendations confirm that these benefits are real but disorder‐specific, classifier‐specific, and dependent on appropriate interpretation (Kerkhof et al. 2024; Husson et al. 2024; Trajkova et al. 2024).

It is important to distinguish episignature classification from locus‐specific methylation testing. Classical clinical methylation assays are used for imprinting disorders, promoter methylation abnormalities or repeat‐associated methylation at defined loci (Cazalla et al. 2026). Episignature testing uses genome‐wide patterns across many CpGs to classify a sample against reference syndromes. Locus‐specific testing asks whether a known regulatory region has an abnormal methylation state. The informative CpG feature sets used by individual classifiers vary between disorders and analytical platforms and are selected from the hundreds of thousands of loci interrogated by genome‐wide methylation arrays. Episignature testing asks whether the overall methylation profile resembles a recognised disorder‐associated pattern. Both approaches are clinically valuable, but they answer different questions.

The interpretive power of an episignature depends on validation. Some signatures are strong, reproducible and specific across independent cohorts; others are weaker, variant‐class‐dependent or less sensitive. A positive, well‐validated episignature can provide compelling functional evidence supporting a molecular diagnosis, although the evidentiary weight assigned to such findings depends on the specific disorder, assay validation and clinical context (Husson et al. 2024; Tkemladze et al. 2026). A negative result is more difficult to interpret because it may reflect the absence of disease, tissue specificity, age‐related effects on methylation patterns, low‐level mosaicism, a hypomorphic variant, a variant mechanism not represented in the training data, or limited sensitivity of the signature. (Tkemladze et al. 2026). In clinical practice, testing is most commonly performed after infancy, as age‐related methylation changes during the neonatal period may complicate interpretation and can potentially reduce the clarity of some disease‐associated methylation signatures.

## 4 Laboratory Generation of CpG Methylation Data

Clinical methylation profiling usually begins with genomic DNA extracted from peripheral blood, although saliva, fibroblasts, or other tissues may be used in selected contexts. In array‐based workflows, DNA undergoes bisulfite conversion. Unmethylated cytosines are deaminated and are subsequently read as thymine, while 5‐methylcytosine remains protected; importantly, standard bisulfite chemistry also leaves 5‐hydroxymethylcytosine indistinguishable from 5‐methylcytosine, so conventional arrays measure bisulfite‐resistant cytosines rather than pure 5mC alone. The converted DNA is hybridised to probes targeting CpG sites across the genome. Fluorescent signal intensities are then used to estimate the proportion of methylated and unmethylated DNA at each measured CpG.

The most intuitive output is the beta value, often written as β = M/(M + U + α), where M is the methylated probe signal, U is the unmethylated probe signal, and α is an offset, commonly 100, used to stabilise the estimate when signal intensities are low. Beta values range from 0 to 1 and are easy to interpret as approximate methylation proportions. For statistical modelling, M‐values are preferred. M‐values represent the log ratio of methylated to unmethylated signal: M‐value = log_2_ (M/U). The log2 transform is used because it centres equal methylated and unmethylated signals at 0, making two‐fold changes symmetric in opposite directions. Beta values are usually preferred for interpretation and visualisation, whereas M‐values are often preferred for statistical testing.

Before clinical interpretation, methylation data undergo quality‐control and analytical processing to minimise technical artefacts and improve reliability. Analytical pipelines are designed to account for sources of biological and technical variation, including age, sex, laboratory batch effects and differences in blood cell composition. These measures help ensure that observed methylation patterns reflect disease‐associated biology rather than technical noise or normal variation between samples.

## 5 Statistical Interpretation of Methylation Episignatures

Episignature analysis is a pattern‐recognition problem applied to high‐dimensional methylation data (Figure 3). Each sample has methylation values across hundreds of thousands of CpG sites, while rare‐disease cohorts may contain only a small number of confirmed cases. This imbalance creates a statistical challenge where the number of variables greatly exceeds the number of samples. Episignature interpretation is built from multiple analytical steps, each addressing a specific aspect of the methylation data. These range from technical quality assessment and identification of informative CpGs to visualisation, classification and clinical validation. Understanding the purpose of each method helps clinicians interpret methylation reports and appreciate the strengths and limitations of episignature‐based diagnostics. Table 2 summarises the principal statistical approaches used in methylation analysis and the clinical questions they are designed to answer.

**FIGURE 3:**
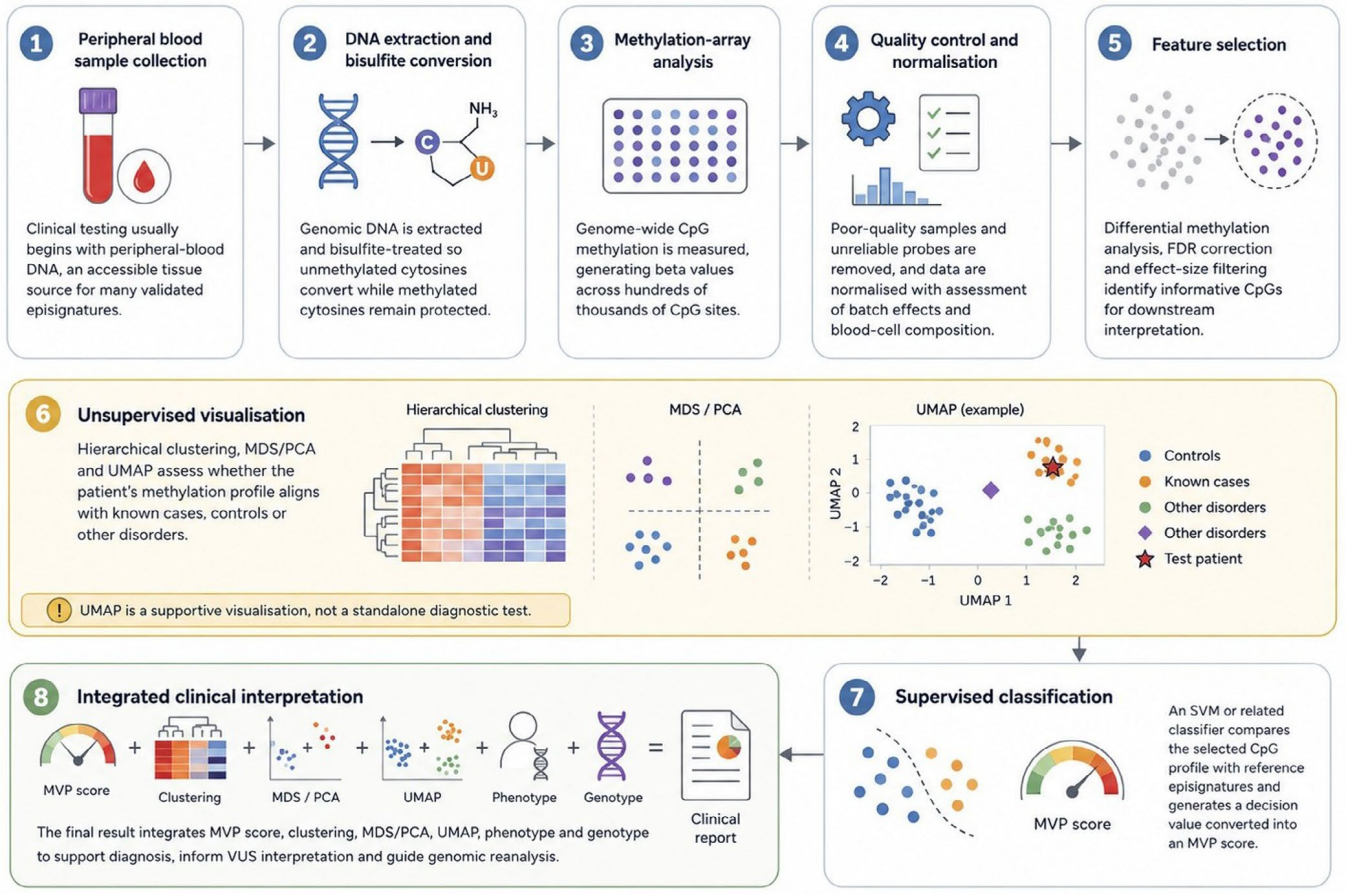
Analytical workflow for episignature testing. Peripheral‐blood DNA undergoes bisulfite conversion, methylation‐array profiling, quality control, normalisation and feature selection. Informative CpGs are then assessed using unsupervised visualisation, including hierarchical clustering, MDS/PCA and UMAP, followed by supervised classification using platform‐specific machine‐learning methods, with classifier‐derived scores such as the MVP score in the EpiSign framework. Final interpretation integrates classifier output, visual clustering, phenotype and genotype. UMAP is used as a supportive visualisation, not as a standalone diagnostic test.

**TABLE 2:** Statistical methods used in methylation episignature analysis and the clinical questions they answer.

Method/concept	When it is used	Clinical question answered
Beta value	Initial review of CpG methylation data and heatmaps	How methylated is this CpG site?
M‐value	Regression‐based modelling and differential methylation analysis	How should methylation be represented for statistical testing?
Quality control and normalisation	Before biological or diagnostic interpretation	Is the methylation dataset technically reliable?
Covariate adjustment	Discovery studies and mixed clinical datasets	Could apparent signal reflect age, sex, batch or blood‐cell composition?
Differential methylation analysis	Discovery or refinement of an episignature	Which CpGs differ reproducibly between cases and controls?
FDR correction	Genome‐wide CpG‐level testing	Which CpGs remain credible after multiple testing?
Feature selection	Reducing CpGs before clustering/classification	Which CpGs best distinguish cases, controls and other disorders?
Clustering and heatmaps	Visual review of a suspected signature	Does the patient cluster with known cases?
MDS/PCA	Visualising high‐dimensional methylation relationships	Do cases, controls and other disorders separate visually?
UMAP visualisation	After feature selection, alongside heatmap clustering and MDS/PCA.	Does the test patient cluster with cases, controls or an intermediate/outlier group?
SVM or related classifier	Diagnostic classification against reference profiles	Which known episignature does the patient most resemble?
MVP score (EpiSign)	Platform‐specific reporting of EpiSign classifier output	How strongly does the patient match the reference episignature?
Sensitivity and specificity	Validation and clinical interpretation	How much confidence should be placed in positive or negative results?
Rare DMR analysis	Unresolved cases, imprinting, promoter methylation, repeats or structural variation	Is there a focal methylation abnormality rather than a known syndrome‐wide pattern?

Differential methylation analysis is usually the first analytical step when an episignature is being developed or refined from a cohort of molecularly confirmed cases and appropriately matched controls. The aim is to identify CpG sites where methylation differs consistently between affected and unaffected individuals, forming the initial set of candidate loci that may contribute to a disorder‐specific methylation pattern. This comparison is performed at each CpG site across the array. In practice, methylation levels are often analysed using linear modelling, with adjustment for relevant covariates. Covariate adjustment means including factors such as age, sex, technical batch and estimated blood‐cell composition in the statistical model so that methylation differences attributable to the disorder can be distinguished from differences caused by biological variation or technical artefact. CpG sites that show reproducible case–control methylation differences are called differentially methylated probes. When several neighbouring CpGs show a coordinated methylation change in the same genomic region, they may define a differentially methylated region. Because methylation arrays test hundreds of thousands of CpG sites simultaneously, some apparent differences will occur by chance. Therefore, multiple‐testing correction is required to reduce false‐positive findings.

Statistical significance alone is insufficient for clinical classification. A CpG with a large, reproducible methylation difference is optimal for distinguishing a disorder from controls. A CpG may reach statistical significance despite a modest effect size when within‐group variability is low; conversely, a large methylation difference may fail stringent significance thresholds in a very small rare‐disease cohort. Therefore, selecting a clinically‐relevant methylation profile requires consideration of effect size, reproducibility, within‐group consistency, separation from other disorders and discriminatory performance. The selected CpGs become the informative feature set used for heatmaps, multidimensional scaling and supervised classification.

The selected informative CpGs, including probes within relevant DMRs, are then used for unsupervised visualisation. Hierarchical clustering groups samples according to methylation similarity across these selected features, and the output is often displayed as a heatmap with a dendrogram. In a strong episignature, affected individuals cluster together and are separate from controls and other syndromes. Multidimensional scaling, principal component analysis and UMAP provide complementary visualisations by reducing high‐dimensional methylation relationships into two or three dimensions. UMAP is a nonlinear dimensionality‐reduction method that can be useful when larger reference datasets include multiple related or partially overlapping disorder groups because it may display local neighbourhood structure and cluster separation more clearly than linear projections. However, UMAP is sensitive to feature selection, scaling, nearest‐neighbour parameters and batch effects and should be interpreted as visual support rather than independent diagnostic evidence. These plots do not diagnose the patient independently. Their purpose is to show whether the patient's methylation profile visually aligns with known cases, controls or an intermediate/outlier group.

Support vector machines and related supervised classifiers perform the diagnostic prediction step using the same informative methylation features. In a simplified linear model, each sample is represented as a vector of methylation values at selected CpGs, including CpGs that capture relevant DMRs. The classifier learns a boundary that separates the disease class from controls or other disorder classes. When a new patient sample is analysed, the model calculates the sample's position relative to that boundary. Supervised classification can be implemented using different machine‐learning approaches. In the EpiSign framework, SVM‐based classification generates decision values that can be transformed into an MVP score, whereas other analytical platforms may use different classifiers and reporting metrics. The MVP score is not a *p*‐value, a measure of methylation burden, an indicator of disease severity, or a direct pathogenicity category under the American College of Medical Genetics and Genomics and Association for Molecular Pathology (ACMG/AMP) framework. It is a classifier‐derived confidence measure of similarity to a reference episignature. A high score is most useful when supported by concordant clustering, dimensionality‐reduction visualisation such as MDS or UMAP, phenotype, genotype and known validation of the signature. Intermediate scores require caution. Negative scores are informative only in relation to the validated sensitivity of the assay for the relevant disorder, variant class, tissue and biological mechanism.

Validation statistics determine how much weight clinicians should place on an episignature result. Sensitivity describes how often individuals with the disorder are detected. Specificity describes how often unaffected individuals and individuals with other disorders test negative. Positive predictive value and negative predictive value depend on the clinical setting and prior probability. Independent validation is especially important because small, rare‐disease discovery cohorts are vulnerable to overfitting. A methylation pattern that separates a small number of molecularly confirmed cases from controls in the initial discovery cohort may not perform with the same accuracy in a broader clinical cohort.

## 6 Computational Tools for Episignature and Multi‐Omic Analysis

A range of open‐source and proprietary computational tools support genome‐wide DNA methylation analysis, although no single software package reproduces the complete workflow of every clinical episignature laboratory. For Illumina methylation arrays, widely used open‐source Bioconductor packages include minfi, SeSAMe and ChAMP (Aryee et al. 2014; Tian et al. 2017; Zhou et al. 2018). minfi provides a flexible and widely used framework for preprocessing and quality control, SeSAMe emphasises probe‐level quality control and artefact reduction, and ChAMP offers a more integrated workflow for preprocessing and downstream analysis. Differential methylation can be modelled using limma, while regional changes can be assessed using DMRcate (Peters et al. 2015). These tools are transparent and flexible, but results can vary according to normalisation, probe filtering, covariate adjustment and other analytical choices.

These generic tools should be distinguished from disorder‐specific classifier platforms. EpiSign is a clinically implemented assay supported by curated reference cohorts and a validated classification framework (Aref‐Eshghi, Bend, et al. 2019, 2020; Sadikovic et al. 2021), whereas EpigenCentral is a freely accessible platform for methylation analysis and classification of rare neurodevelopmental disorders (Aref‐Eshghi et al. 2020; Awamleh et al. 2022; Turinsky et al. 2020). Their reference datasets and classifier implementations differ, and their outputs should not be assumed to be directly interchangeable.

For multi‐omic integration, MOFA2 provides unsupervised integration across molecular data types, whereas mixOmics/DIABLO supports supervised integration and feature selection (Argelaguet et al. 2018; Singh et al. 2019). MOFA2 is useful for exploratory analysis but does not produce disease‐specific classifiers, while DIABLO can identify discriminatory multi‐omic features but may overfit small rare‐disease cohorts. Commercial tools such as QIAGEN Ingenuity Pathway Analysis can aid pathway interpretation but are proprietary. Overall, open‐source tools offer transparency and flexibility, whereas clinically implemented or commercial platforms provide greater standardisation but less methodological transparency.

## 7 Approach to CpG Methylation Data Analysis

A structured analytical framework is used to derive episignatures from CpG methylation data, moving from raw beta values through feature selection and pattern recognition to supervised classification (Figure 4). The workflow integrates differential methylation assessment, unsupervised clustering, dimensionality reduction, and machine‐learning–based classification to contextualise the test sample relative to controls, affected individuals, and comparator disorders.

**FIGURE 4:**
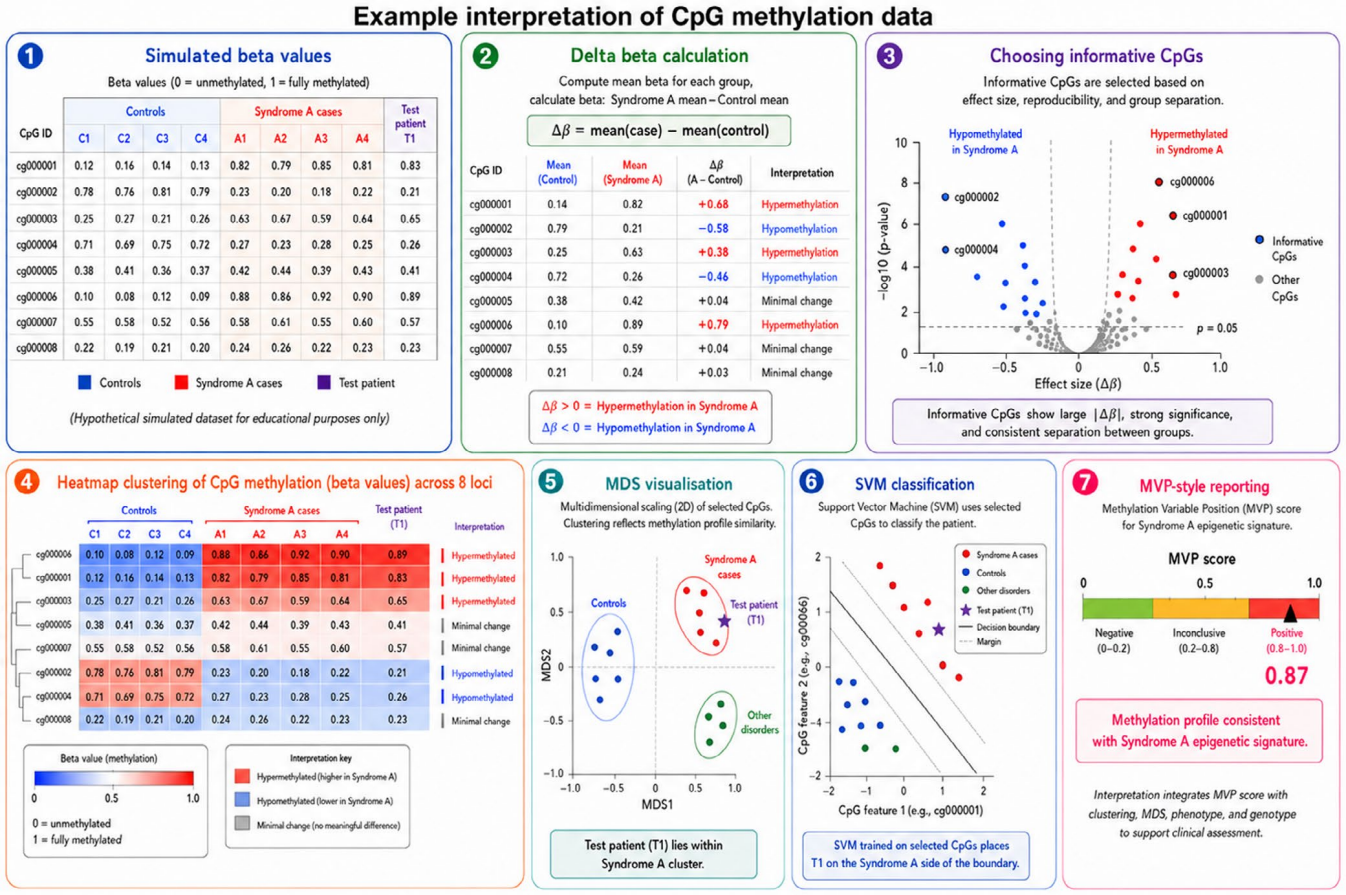
Example interpretation of CpG methylation data. (1) A CpG methylation matrix shows beta values for unaffected controls, Syndrome A cases and a test patient. (2) Group mean beta values and delta beta values, calculated as mean methylation in affected cases minus mean methylation in unaffected controls, demonstrate relative hypermethylation and hypomethylation. (3) Informative CpGs are selected using effect size, statistical support, and reproducible separation. (4) Selected CpGs are used for heatmap clustering. (5) Multidimensional scaling (MDS) displays methylation similarity in two dimensions. (6) Supervised classification places the test patient relative to reference samples. (7) Platform‐specific classifier output is generated; for example, the EpiSign framework reports an MVP score.

The analysis begins with a beta‐value matrix comprising controls, molecularly confirmed affected individuals and the test patient, where each row represents a CpG site and each column represents a sample. From this matrix, group‐level mean beta values are derived and used to calculate delta beta values, identifying CpGs with consistent case–control differences. For example, a CpG with a mean beta value of 0.19 in controls and 0.80 in affected individuals yields a delta beta of +0.61, indicating relative hypermethylation in cases, whereas a control mean of 0.73 versus 0.27 in cases yields a delta beta of −0.46, indicating hypomethylation.

Informative CpGs are then selected based on effect size, statistical significance, and reproducibility of separation between groups. These selected features are visualised using hierarchical clustering, typically as a heatmap with dendrogram structure, where robust episignatures show consistent clustering of affected individuals separate from controls and comparator disorders. The position of the test patient relative to these clusters provides an initial qualitative assessment of concordance. Multidimensional scaling (MDS) provides a complementary representation of the same relationships by projecting pairwise distances between samples into a low‐dimensional space. Samples with similar methylation profiles cluster together, whereas dissimilar profiles separate. The test sample's proximity to the affected cluster provides visual corroboration of the clustering result, but MDS is not used as a standalone diagnostic classifier.

Supervised classification is then performed using the selected CpGs as feature inputs and labelled reference cohorts. In models such as support vector machines, each sample is represented as a vector in high‐dimensional methylation space, and the algorithm learns a decision boundary separating disease‐associated from non‐disease profiles. The test sample is projected into this model to generate a decision value (methylation variant pathogenicity (MVP) score) reflecting its relative position to the learned boundary.

The MVP score is typically scaled between 0 and 1. Higher values indicate stronger concordance with the episignature, whereas lower values argue against it. The MVP score should be interpreted strictly as a measure of classifier‐derived similarity rather than as a statistical probability, disease severity metric, or ACMG/AMP pathogenicity class.

A robust positive episignature is supported by concordant CpG‐level differences, reproducible clustering, consistent MDS positioning, a supportive classifier/MVP score, and coherence with the patient's clinical and genetic context. Discordant findings should prompt reassessment of technical quality, tissue specificity, mosaicism, reference cohort composition, and known limitations of the episignature model.

## 8 Current Genomic and Epigenomic Testing Approaches in GDD/ID and Rare Disease

The evaluation of developmental delay, intellectual disability and rare disease increasingly requires a tiered and mechanism‐aware approach (Figure 5). No single test detects all classes of pathogenic variation. Instead, chromosomal microarray, exome sequencing, genome sequencing, long‐read sequencing, RNA sequencing and DNA methylation profiling each interrogate different biological mechanisms and provide distinct forms of diagnostic evidence. Understanding the strengths and limitations of each modality is essential for selecting appropriate investigations and interpreting results in unresolved cases.

**FIGURE 5:**
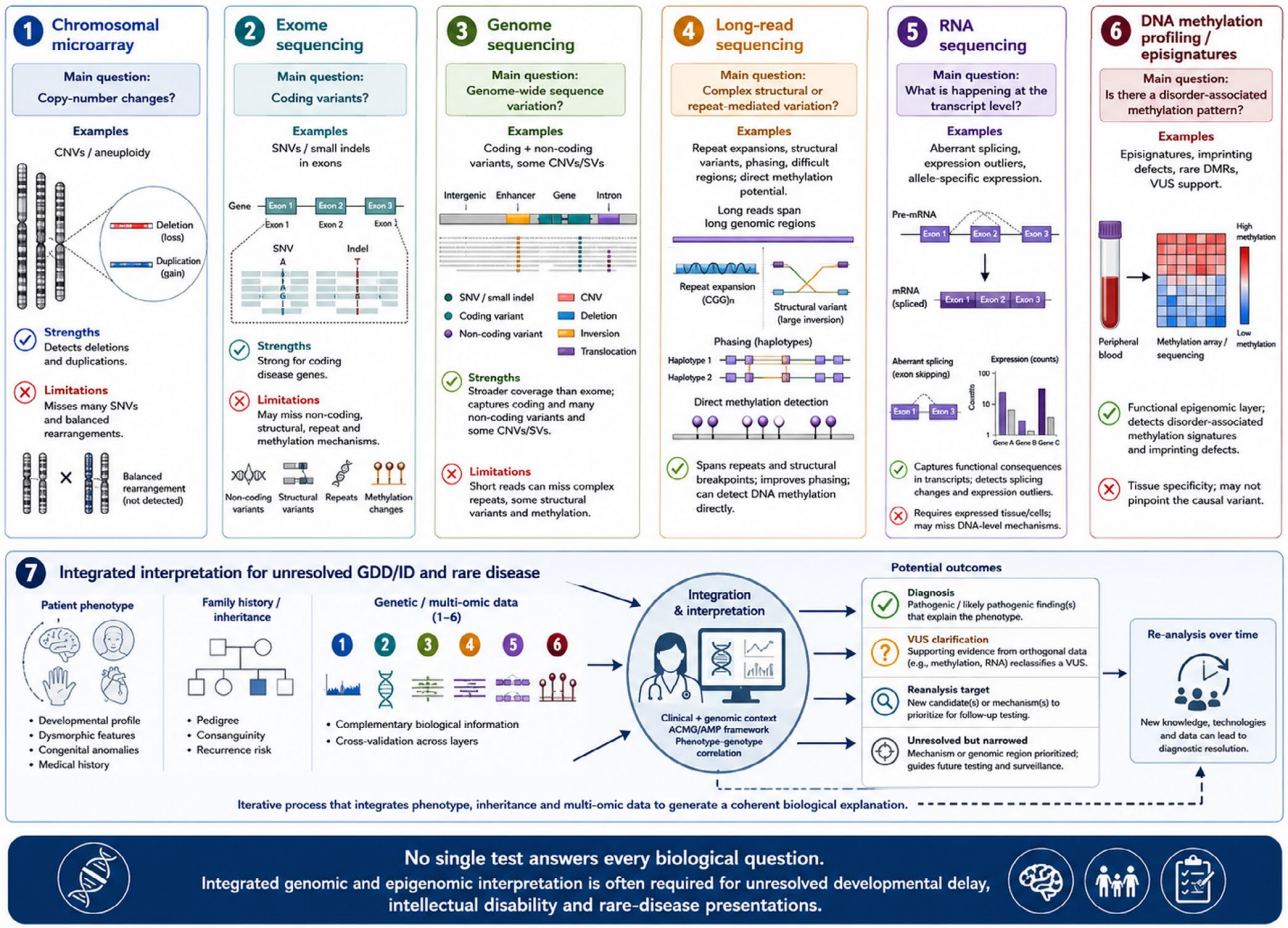
Position of epigenomic testing in rare‐disease diagnostics. Chromosomal microarray, exome sequencing, genome sequencing, long‐read sequencing, RNA sequencing, and DNA methylation profiling answer different biological questions. Integrated interpretation of phenotype, inheritance, genomic data, and functional multi‐omic evidence is often required for unresolved developmental delay, intellectual disability, and rare‐disease presentations.

Chromosomal microarray remains an important tool for detecting pathogenic copy‐number variants, including deletions, duplications, and aneuploidies. Although it does not detect most single‐nucleotide variants, balanced rearrangements, or epigenetic abnormalities, it continues to provide robust diagnostic yield in disorders where copy‐number variation is a major contributor to disease.

Exome sequencing focuses on the protein‐coding regions of the genome, where a large proportion of known disease‐causing variants reside. It is highly effective for detecting coding single‐nucleotide variants, canonical splice variants, and small insertions or deletions, but has more limited sensitivity for non‐coding variants, structural variation, repeat expansions, and epigenetic mechanisms.

Genome sequencing extends analysis beyond coding regions and provides more uniform genomic coverage. In addition to identifying coding variants, it enables assessment of non‐coding variation, mitochondrial DNA variants, and selected structural variants, making it an increasingly important first‐line test for unresolved rare disease.

Long‐read sequencing is an important emerging technology for unresolved rare diseases. Long reads can span repetitive and structurally complex regions, improve detection of structural variants, resolve haplotypes and identify repeat expansions that may be missed by short‐read approaches (Del Gobbo and Boycott 2025). Depending on platform and analysis, long‐read methods can also provide direct information about DNA methylation.

RNA sequencing occupies a complementary position. It can identify aberrant splicing, expression outliers, monoallelic expression and consequences of non‐coding or synonymous variants that are difficult to interpret from DNA sequence alone (Liu et al. 2026). Its clinical utility is constrained by tissue availability, gene expression in the sampled tissue, allelic imbalance and the instability of some aberrant transcripts due to nonsense‐mediated decay. Nevertheless, RNA sequencing can be decisive for splice variants, deep intronic variants and expression‐altering regulatory variants.

DNA methylation arrays provide a different kind of evidence. They do not detect all causal variants, but they can show that the genome has adopted a methylation profile consistent with a known disorder or that a local genomic region has abnormal methylation. This makes methylation testing useful for variant of uncertain significance (VUS) interpretation and unresolved cases in suspected chromatinopathies, imprinting disorders, promoter methylation defects and repeat‐associated methylation changes. In clinical practice, methylation testing should be positioned alongside sequencing and transcriptomics, not as a replacement for them (Aref‐Eshghi, Bend, et al. 2019, 2020, 2019; Sadikovic et al. 2021; Kumps et al. 2023; Ivashchenko et al. 2026; Levy et al. 2022; Kerkhof et al. 2022).

## 9 Clinical Applications of DNA Methylation and Epigenomic Testing

The most established clinical application of episignature testing is the interpretation of variants of uncertain significance in genes with validated methylation signatures. If a patient has a VUS in *KMT2D, NSD1, DNMT3A, KDM5C* or another episignature‐associated gene, a concordant methylation profile can provide functional evidence that the variant perturbs the relevant disease pathway (Husson et al. 2024; Niceta et al. 2024; Testa et al. 2023). When integrated with phenotype, segregation, population frequency, computational prediction and other functional data, this evidence may contribute to variant reclassification.

A negative episignature result can reduce support for pathogenicity, but only with caution. Its interpretive value depends on multiple factors, including the robustness and sensitivity of the gene‐specific signature, representation of the relevant variant class within training cohorts, the expectation that the disease mechanism produces a detectable blood‐based signature, and the possibility of mosaicism. Diagnostic performance may also be influenced by the composition of the reference dataset, including the representation of different ancestral backgrounds and age groups. In some disorders, methylation signatures may vary with developmental stage, and testing at an age when the signature is incompletely established may reduce sensitivity. Furthermore, although the effects of acute illness, inflammation and other physiological stressors on episignature performance remain incompletely understood, these factors may influence blood‐based methylation profiles and warrant further investigation. For some disorders, truncating variants produce a readily detectable signature, whereas missense, hypomorphic or domain‐specific variants may generate weaker, atypical or absent profiles. Negative methylation findings should therefore not be interpreted automatically as evidence for benignity.

Beyond variant classification, episignatures can distinguish clinically overlapping disorders and refine molecular diagnoses (Pavinato et al. 2025). Many chromatinopathies present with developmental delay, intellectual disability, epilepsy, growth abnormalities, congenital anomalies and dysmorphic features, with substantial phenotypic overlap between syndromes. In such situations, a methylation profile may identify a syndrome not initially suspected or redirect genomic reanalysis towards an alternative diagnosis. Importantly, episignatures can also stratify biologically distinct disease mechanisms within a single gene or across related genes. A notable example is Menke–Hennekam syndrome, caused by specific variants in *CREBBP* and *EP300*. Genome‐wide methylation profiling has identified distinct domain‐specific episignatures associated with variants affecting the ZZ, TAZ2 and ID4 regions of these proteins, demonstrating that methylation signatures can distinguish molecular subtypes that are mechanism‐specific rather than gene‐specific (Haghshenas et al. 2024). This example illustrates how epigenomic profiling can reveal underlying biological heterogeneity, refine genotype–phenotype correlations and support clinically meaningful subclassification of rare disorders.

In unresolved cases, broad methylation screening may identify a known episignature even when the causal variant was missed by previous testing. Methylation analysis may also detect rare differentially methylated regions (DMRs) suggestive of imprinting disturbances, promoter silencing, copy‐number‐associated methylation changes, repeat expansions, or structural rearrangements.

Epigenomic testing can also contribute to family studies and reproductive counselling. A positive episignature may clarify whether a variant is likely to be disease‐causing in the proband and can inform targeted testing in relatives. In some X‐linked disorders, methylation signatures may assist in the assessment of carrier states or variable expression, although interpretation remains syndrome‐specific and evidence‐based. For mosaic disorders, methylation profiles may provide supportive evidence, but sensitivity depends on the affected tissue distribution and the proportion of cells carrying the pathogenic alteration.

## 10 Limitations and Interpretive Cautions

Episignature testing is a powerful functional diagnostic tool, but its results must be interpreted within defined biological and technical limits. Its principal limitation is that only a subset of rare‐disease genes and disorders currently have validated DNA methylation episignatures. Most genetic disorders do not yet have validated DNA methylation episignatures, and some disorders may never produce a detectable signature in peripheral blood. Therefore, a negative episignature screen does not exclude a genetic or epigenetic diagnosis. It only indicates that the tested sample did not match the reference signatures included in the assay, or that no reportable abnormality was detected under that laboratory's diagnostic approach.

Tissue specificity is another major limitation. Peripheral blood is practical and clinically accessible, but many neurodevelopmental disorders primarily affect the brain. Some disorders generate robust blood‐detectable episignatures, whereas others may exhibit methylation changes that are restricted to neural tissue, particular cell populations, or specific developmental age windows. As a result, both the tissue sampled and the age at testing may influence the detectability of a methylation signature. Alternative tissues such as saliva, fibroblasts or disease‐relevant tissue may occasionally provide additional information, but most reference datasets and validated episignatures have been derived from peripheral blood, making cross‐tissue interpretation challenging.

Mosaicism can also weaken or obscure an episignature. If the pathogenic variant is present in only a proportion of blood cells, the resulting methylation profile may be attenuated and may fall below the reporting threshold. Mosaicism confined to tissues outside blood may be missed entirely by a blood‐based assay. Similarly, hypomorphic variants, domain‐specific missense variants, splice‐altering variants with residual function, or variants affecting only selected isoforms may produce partial, atypical, or intermediate molecular profiles (Oexle et al. 2023).

Episignature interpretation is also dependent on the quality and scope of the reference database. Machine‐learning classifiers require well‐characterised training samples with confirmed molecular diagnoses. Small training cohorts, limited ancestral diversity, batch effects, technical platform differences, and biological overlap between related disorders can all influence sensitivity and specificity.

Finally, episignatures do not replace careful clinical assessment. DNA methylation results should be integrated with phenotype, inheritance pattern, variant mechanism, segregation data, and other laboratory evidence. A positive episignature that is discordant with the phenotype or genotype should prompt reanalysis rather than automatic diagnostic acceptance. Conversely, a negative result should not override strong clinical or genetic evidence when the suspected disorder is not well represented in the assay, has limited sensitivity, or may not generate a detectable blood‐based signature. In practice, episignature testing is best viewed as an additional layer of functional evidence, not as a standalone diagnostic endpoint.

## 11 Emerging Directions

Clinical epigenomics is moving from a specialised diagnostic adjunct into a broader functional genomics framework. DNA methylation episignatures are currently the most mature clinical application, particularly in neurodevelopmental disorders and chromatinopathies. Future developments are likely to centre on several interconnected areas: expansion of episignature reference datasets, integration with long‐read sequencing, multi‐omic interpretation, and emerging epitranscriptomic technologies.

A major priority is the continued expansion of episignature reference databases. Larger and more diverse cohorts will facilitate the discovery of new disorder‐specific signatures, improve the sensitivity and specificity of existing classifiers, and increase confidence in clinical interpretation. Importantly, reference datasets must become more ancestrally, geographically and clinically representative to minimise bias and ensure equitable diagnostic performance across populations. Prospective studies are also needed to determine the impact of methylation testing on diagnostic yield, variant interpretation, clinical management, reproductive counselling and health‐economic outcomes.

A related challenge is extending episignatures beyond the tissue in which they were originally developed. Reko and colleagues recently used machine‐learning approaches to transform a blood‐derived Down syndrome (MIM #190685) episignature into cell‐type‐agnostic classifiers that performed across six tissue types. By prioritising methylation features that remain informative across tissues, this proof‐of‐concept approach enabled classification of prenatal amniotic and placental samples. Such strategies may provide a route for adapting established blood‐derived episignatures to prenatal and other non‐blood tissues, directly addressing the tissue specificity that currently limits broader clinical application (Reko et al. 2026).

Long‐read sequencing is the technology most likely to reshape clinical epigenomics in the near term. Native DNA long‐read platforms, particularly nanopore sequencing, can simultaneously detect sequence variants, structural variants, repeat expansions, haplotypes, and DNA methylation from the same molecule without bisulfite conversion. This integrated approach can reveal mechanisms missed by short‐read sequencing, including complex structural rearrangements, repeat‐mediated disorders, allele‐specific methylation, and variation within difficult‐to‐map regions. The principal advantage of long‐read sequencing extends beyond variant detection to contextual interpretation. Phasing can establish whether sequence variants and methylation changes occur on the same allele, while direct resolution of repeat expansions and structural variants can reveal genomic mechanisms underlying altered methylation patterns. However, widespread clinical implementation will require further advances in analytical standardisation, variant‐ and methylation‐calling performance, cost‐effectiveness, throughput, and integrated reporting frameworks (Del Gobbo and Boycott 2025; Geysens et al. 2025; Smits et al. 2026).

Another important direction is multi‐omic integration. DNA methylation, RNA sequencing, chromatin accessibility, proteomics and phenotype‐driven computational analysis each contribute complementary functional evidence (Audet et al. 2023; Krgovic et al. 2026). A variant of uncertain significance may be reclassified when one or more of these approaches demonstrate a measurable molecular consequence, such as a characteristic episignature, aberrant splicing, reduced transcript abundance, allele‐specific expression or perturbation of a disease‐relevant pathway (Kerkhof et al. 2024; Aref‐Eshghi et al. 2020; Liu et al. 2026). The central interpretive challenge will be determining which data layers provide independent evidence, which are biologically redundant, and how to combine them in variant classification and diagnostic reporting (Yang et al. 2025). Efforts to categorise methylation signature information as functional evidence are ongoing (Koparir et al. 2026).

DNA methylation is currently the most clinically mature epigenomic biomarker, but other genome‐wide regulatory states can also generate disease‐associated molecular signatures. These include 5‐hydroxymethylcytosine profiles, histone‐modification landscapes, chromatin‐accessibility patterns, nucleosome positioning and higher‐order chromatin organisation. For example, ATAC‐seq identifies regions of accessible chromatin and can reveal altered regulatory‐element activity (Buenrostro et al. 2013), whereas chromatin‐profiling approaches such as CUT&Tag can map histone modifications and chromatin‐associated proteins (Kaya‐Okur et al. 2019). Distinguishing 5‐hydroxymethylcytosine from 5‐methylcytosine may also provide information that is obscured by conventional bisulfite‐based methylation arrays. Transcriptomic expression and splicing profiles can similarly form reproducible molecular signatures, although these are more appropriately considered transcriptomic rather than epigenomic biomarkers. At present, these approaches remain less standardised and more dependent on tissue, cell type and sample handling than peripheral‐blood DNA methylation episignatures, and few have undergone comparable clinical validation for constitutional rare disease. Their near‐term role is therefore likely to be complementary functional investigation and multi‐omic integration rather than standalone diagnostic classification.

Epitranscriptomics represents another emerging layer of molecular signature analysis. RNA modifications, particularly N6‐methyladenosine (m6A), extend gene regulation beyond DNA sequence, chromatin state and transcript abundance. As the most abundant internal modification of eukaryotic messenger RNA, m6A influences RNA splicing, localisation, stability, translation and decay through a writer–eraser–reader system analogous to that seen in DNA and histone regulation.

m6A biology is of particular interest in rare disease because many neurodevelopmental disorders arise from disruption of gene regulation rather than alterations in protein sequence alone. Changes in RNA modification may influence transcript abundance, isoform usage, RNA processing and translation efficiency, potentially contributing to disease mechanisms that are not readily apparent from DNA sequence analysis. m6A also regulates non‐coding RNAs, including long non‐coding RNAs, circular RNAs and microRNAs, further expanding the complexity of post‐transcriptional gene regulation (Doijad et al. 2026; Yi et al. 2025). These modifications can alter RNA stability, localisation, RNA‐binding protein interactions, competing endogenous RNA behaviour and, in some contexts, small‐peptide coding potential. These mechanisms are being explored as biomarkers and therapeutic targets, especially in cancer biology, but their role in routine rare‐disease diagnostics remains investigational (Yi et al. 2025).

At present, however, epitranscriptomic profiling remains a translational research tool rather than a validated clinical diagnostic assay. Unlike DNA methylation episignatures, m6A profiling lacks standardised clinical workflows, disease‐specific reference datasets and established frameworks for variant interpretation. Although advances in sequencing technologies continue to improve the detection and quantification of RNA modifications, their role in routine rare‐disease diagnostics remains investigational (Eladl et al. 2026).

The broader trend is a shift from single‐modality testing towards integrated functional genomics. Chromatin state influences transcriptional potential, DNA methylation and histone modifications shape gene regulation, RNA processing and modification determine transcript fate, and translational mechanisms ultimately influence protein output. Together, these layers provide complementary insights into how genetic variation produces disease.

For the foreseeable future, DNA methylation episignatures will remain the most clinically mature epigenomic application. However, integration with genome sequencing, long‐read sequencing, RNA sequencing and other functional assays is likely to provide a more complete understanding of disease mechanisms than any individual technology alone. In unresolved rare disease, combining genomic, epigenomic and transcriptomic evidence may improve diagnostic resolution, facilitate variant interpretation and reduce the risk of over‐interpreting isolated findings. As these technologies mature, rare‐disease diagnostics is likely to move increasingly from variant detection towards integrated interpretation of genotype, molecular function and phenotype.

## 12 Conclusion

Clinical epigenomic diagnostics can serve as a functional adjunct to rare‐disease evaluation by assessing the downstream regulatory consequences of genomic variation. Germline DNA methylation episignatures are currently the most mature clinical application, particularly in chromatinopathies and neurodevelopmental disorders. Rather than identifying the causal variant itself, episignatures provide evidence of its downstream biological consequences, helping to support diagnosis, resolve selected variants of uncertain significance, distinguish clinically overlapping syndromes and guide reanalysis of previously unsolved cases.

Effective interpretation requires an understanding of both the biology and analytics underpinning epigenomic testing. Episignatures arise from disruption of epigenetic regulatory networks involving DNA, histone, and RNA modifications and are interpreted through a combination of statistical, visualisation, and machine‐learning approaches. Their clinical utility must also be considered within the context of important limitations, including tissue specificity, developmental effects, mosaicism, incomplete disorder coverage, reference‐dataset dependence, and variable assay sensitivity.

Looking forward, the greatest opportunity may lie in integrating epigenomic data with other functional and genomic modalities. DNA methylation profiling, RNA sequencing, long‐read sequencing, chromatin accessibility assays, proteomics and phenotype‐driven computational analysis each provide complementary insights into disease mechanisms. Together, these approaches have the potential to move rare‐disease diagnostics beyond variant detection towards a more comprehensive understanding of how genomic variation perturbs gene regulation and ultimately produces disease. As integrated multi‐omic testing becomes increasingly accessible, the distinction between genomic and epigenomic diagnostics is likely to blur, creating a more unified framework for connecting genotype, molecular function and phenotype.

## Author Contributions

**Himanshu Goel:** conceptualization, writing – original draft, methodology, writing – review and editing. **Shuxiang Goh:** writing – review and editing. **Luke Stuart:** writing – review and editing.

## Funding

The authors have nothing to report.

## Ethics Statement

Ethical approval was not required because this review does not report new human participant data, identifiable patient information, animal experiments or interventional research. Hypothetical examples and simulated datasets are included for educational purposes only.

## Conflicts of Interest

The authors declare no conflicts of interest.

## Data Availability

No new patient‐level or experimental datasets were generated for this review. Any supplementary methylation data used for educational purposes are simulated and should not be used for biological inference.
